# Differences in Tooth Development in Patients With Unilateral Cleft Lip and Palate

**DOI:** 10.1002/cre2.70086

**Published:** 2025-02-22

**Authors:** Marie Schwarting, Heinrich Wehrbein, Irene Schmidtmann, Christina Erbe, Susanne Wriedt

**Affiliations:** ^1^ Department of Orthodontics University Medical Center at the Johannes Gutenberg‐University Mainz Germany; ^2^ Institute of Medical Biostatistics, Epidemiology and Informatics (IMBEI) University Medical Center at the Johannes Gutenberg‐University Mainz Germany

**Keywords:** case–control study, cleft lip, cleft palate, dental age, odontogenesis

## Abstract

**Objectives:**

The aim of this study was to investigate tooth development in patients with unilateral cleft lip and palate (UCLP) and unilateral cleft lip and alveolus (UCLA).

**Material and Methods:**

A retrospective case–control study was carried out; 180 panoramic radiographs (OPGs) from non‐syndromic patients (160 UCLP, 20 UCLA) treated at the University Medical Center Mainz (2019–2022) were analyzed. Patients were matched to a control group by calendar age, gender, and ethnicity. Inclusion criteria were verified through clinical data, photographs, and radiographs. No follow‐up was conducted for this study.

**Results:**

The cleft group showed a significantly lower dental age compared to the control group (10.72 ± 2.65 vs. 11.41 ± 2.79; *p* = 0.017), with a mean difference of 0.69 years (95% CI: 0.13–1.25 years). Tooth development was slower on the cleft side (*p* = 0.001), and maxillary teeth lagged behind mandibular teeth (*p* < 0.001). The difference in the control group was somewhat smaller, with a mean difference of 0.11 degrees of mineralization in the control group compared to 0.25 degrees of mineralization in the cleft group. In UCLP patients, significant mineralization differences were noted for the lateral incisors (*p* = 0.004), the central incisor (*p* = 0.047), and canine (*p* = 0.030).

**Conclusions:**

Patients with unilateral clefts show delayed tooth development and dental age, particularly in the affected quadrant. In everyday treatment, attention should be paid to a later start of orthodontic tooth movement to avoid damaging the slower developing roots.

## 1 Introduction

Cleft lip and palate is one of the most common congenital malformations in humans. The incidence is approximately 1:700 (Mossey et al. 2009). Due to the restricted growth of the upper jaw, patients with a cleft usually face a variety of lifelong problems and often have speech, hearing, and dental problems (Singh et al. 2021; Hopkins et al. 2019). Beyond hearing and speech problems, the aesthetic component also plays a major role, which, if the cleft is not treated, has considerable psychological consequences for the patient and his or her family, including reduced quality of life (Shkoukani et al. 2013; Taib et al. 2015). In addition, a higher prevalence of severe dental anomalies is observed; in particular, abnormal tooth formation and hypo‐ and aplasia occur frequently in these patients (Suzuki et al. 1992; Tsai et al. 1998; Tan et al. 2018). Furthermore, tooth developmental delays are also reported, although it is not yet conclusively clarified whether a laterally uneven mineralization of the teeth occurs in cleft lip and palate patients and which teeth are most affected (Tan et al. 2017).

Affected patients require long‐term, interdisciplinary therapy until adulthood. Tooth development is of great clinical importance when planning orthodontic treatment to accurately estimate the time to initiate orthodontic treatment. In addition, the surgical timing for bone graft in affected patients is based on tooth development and not on chronological age (Freitas et al. 2013; Ozawa et al. 2007). Therefore, an accurate assessment of tooth development not only enables better orthodontic and surgical planning with the aim of limiting the number of surgeries required and optimizing treatment but also allows age estimation based on tooth development for forensic purposes (Van Dyck et al. 2021).

The following case–control study aimed to investigate peculiarities in tooth mineralization in patients with cleft formation compared to a group of unaffected patients.

## 2 Material and Methods

### 2.1 Study Design and Setting

This study was designed as a retrospective analysis using panoramic radiographs obtained from the orthodontic archive of the University Medical Center Mainz. Patient recruitment took place between 2019 and 2022. The study was approved by the Ethics Committee of the Medical Chamber of Rhineland Palatinate (approval number 2020‐15539‐retrospective).

### 2.2 Study Population

For patient inclusion, the clinical data and available photographic records of each patient were first reviewed. Following this, the panoramic radiographs were analyzed. Only patients whose clinical data and radiographs met the inclusion criteria were included in the study. The inclusion criteria were Caucasian patients aged between 7 and 14 years, diagnosed with UCLP or UCLA, without craniofacial syndromes. A total of 132 patients were included in the analysis, supplemented by additional radiographs taken from some patients in the same group at a minimum interval of 24 months between two radiographs.

### 2.3 Exclusion Criteria

The exclusion criteria were as follows:

- Inadequate quality of orthopantomograms.
- Cleft types not involving the alveolus.
- Patients diagnosed with craniofacial syndromes.
- Patients older than 14 years or younger than 7 years of age.
- Loss of permanent teeth due to caries or trauma.

A matched control group was established for the 180 radiographs from the cleft group. The control radiographs were also sourced from the archive of the Clinic for Dental, Oral, and Maxillofacial Diseases at the University Medical Center Mainz. A control partner was matched for each cleft patient based on gender and age at the time of the first radiograph. To improve matching accuracy, a subgroup (*n* = 310: 155 cleft, 155 control) was selected from the primary sample (*n* = 360: 180 cleft, 180 control) for sensitivity analysis. This subgroup consisted of pairs that were identical in gender and had an age difference of less than 6 months at the time of the first orthopantomogram. Statistical analyses were performed on both the primary and the matched subgroups.

### 2.4 Sample Size Determination

The sample size was determined based on a power analysis to ensure sufficient statistical power to detect meaningful differences between the groups. Based on available data, we estimated that approximately 180 radiographs of patients with unilateral cleft lip and palate (UCLP) and an equal number of age‐ and sex‐matched controls would be available for analysis. The primary endpoint was defined as dental age according to the methods of Demirjian et al. (1973) and Willems et al. (2001), allowing us to compare patients with clefts to healthy controls. Power analysis showed that with 180 cases and controls, a difference of 0.343 standard deviations (SD) could be detected at a 5% significance level with 90% power, corresponding to a small to medium effect according to Cohen. With 180 radiographs per group, the 95% confidence interval for the prevalence of individual findings would be within 7.5% of the estimated prevalence, with a coverage probability of at least 90%. Exploratory analyses were also planned using the *t*‐test or Wilcoxon test for quantitative variables, depending on the distribution, and χ^2^ testsfor categorical data.

### 2.5 Data Collection and Follow‐Up

Radiographs were collected from the archive by trained persons. The clinical data and photographic records of the patients were reviewed before the radiographs were analyzed. No follow‐up was conducted in this study.

### 2.6 Study Procedures

For the analysis of tooth mineralization, the methods of both Demirjian (Demirjian et al. 1973) and Willems (Willems et al. 2001) were used. The Demirjian et al. (1973) method, extensively used for tooth age determination, categorizes tooth mineralization into 8 stages (A‐H). By examining the mineralization stages of all teeth (excluding wisdom teeth) in the third quadrant and assigning numerical values according to gender, the dental age of a patient can be determined. Willems et al. (2001) later refined this method and developed a more precise table for determining tooth age, as there have been reports of overestimates of the method (Paddenberg et al. 2024). Comparisons between the methods showed that Willems’ approach was less prone to under‐ or overestimation of tooth age.

The following aspects were examined:

i. The degrees of mineralization of all teeth in the third quadrant (excluding wisdom teeth) were determined, and the dental age of each patient was calculated using the Willems tables.
ii. The degrees of mineralization of all maxillary teeth were determined according to Demirjian, and the mean values of the cleft and opposite sides were compared.
iii. All mandibular teeth were determined according to Demirjian, and the mean values of the degrees of mineralization between the upper and lower jaw were compared.

Furthermore, the lateral incisor located on the side of the cleft was examined to determine whether it was erupted mesially or distally, missing, or duplicated. The evaluation was conducted by two independent investigators. In cases of divergent findings, the corresponding analyses were re‐evaluated, discussed, and mutually assessed.

### 2.7 Statistics

The statistical analysis was conducted using SPSS 27 (IBM).

Metric variables were assessed for normal distribution using both the Kolmogorov–Smirnov test and the Shapiro–Wilk test. If a normal distribution was present, metric variables were reported as mean ± 1 standard deviation (mean ± 1 SD). Furthermore, the 95% confidence interval of the mean values was reported (95% CI). Nominal scaled variables were described as absolute and relative frequencies (*n* (%)). For relative frequencies, Clopper–Pearson 95% confidence intervals were also calculated. In order to detect group differences regarding the degree of mineralization of several groups of teeth (cleft side vs. opposite side, maxilla vs. mandible), mean values were calculated for the degree of mineralization of the teeth in question. The mean value was chosen to avoid distortion due to missing teeth. Means of mineralization within patients (e.g., cleft side vs. opposite side, maxilla vs. mandible) were compared using a paired t‐test. Means between patient groups were compared using two‐sample t‐tests. When comparing the degree of mineralization among individual pairs of teeth, the Wilcoxon test was used due to the ordinal scaled variable.

## 3 Results

### 3.1 Patient Demographics

Of the 180 affected patients, 160 (88.9%) had a unilateral cleft lip and palate (UCLP) and 20 (11.1%) had a cleft lip and alveolus (UCLA). Matching of the two groups according to age, gender, and ethnicity resulted in a mean value of 125.0 months (standard deviation: 26.0) in relation to the calendar age in the cleft group and a mean value of 125.6 months (standard deviation: 25.4) in relation to the calendar age in the control group. In both groups, of the total of 180 patients, 123 were males (68.3%) and 57 were females (31.7%).

### 3.2 Dental Maturation: The Cleft Group Versus the Control Group

**Figure 1:**
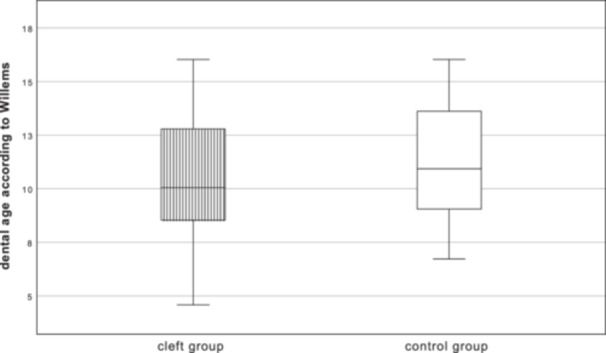
Comparison of the dental age of the cleft group (striped) to the control group (white); mean difference: 0.69 years; *p* = 0.017.

On average, the dental age of the cleft group (*n* = 180) was 10.72 ± 2.65 years, with a 95% CI (10.33–11.11), and that of the control group (*n* = 180) was 11.41 ± 2.79 years, with a 95% CI (11.00–11.82) (see Figure 1, where quartiles are displayed in box plots). The mean difference between the two groups was 0.69 years, with a 95% CI (0.13–1.25). A significant result (*p* = 0.017) was obtained using the two‐sample *t*‐test.

*Patients of the cleft group have a significantly lower dental age than the healthy control group*.

### 3.3 Asymmetric Tooth Formation

**Figure 2:**
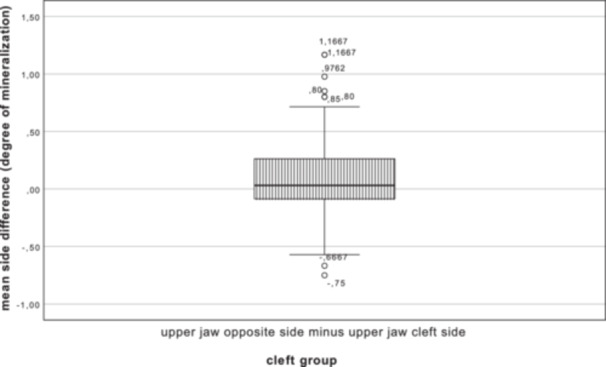
Mean difference between the maxillary cleft side and the opposite side; mean difference: 0.08 degrees of mineralization; *p* = 0.001.

In the cleft group (*n* = 180), the mean values were calculated for both the cleft and the opposite side. The mean value of the affected side was 6.45 ± 0.97 degrees of mineralization, with a 95% CI (6.30–6.59), and that of the opposite side was 6.52 ± 0.99 degrees of mineralization, with a 95% CI (6.38–6.67) (see Figure 2, where quartiles are displayed in box plots). The mean difference was therefore 0.08 degrees of mineralization, with a 95% CI (0.031–0.122). A significant result (*p* = 0.001) was obtained using a paired *t*‐test.

*The mean degree of mineralization of the opposite side is significantly higher than that of the cleft side*.

The Wilcoxon signed‐rank test was used to compare the individual pairs of teeth in the maxilla in the cleft group (*n* = 180). The central incisor (*p* = 0.047), the lateral incisor (*p* = 0.004), and the canine (*p* = 0.030) showed significant differences compared to those of the opposite side.

### 3.4 Upper Versus Lower Jaw

**Figure 3:**
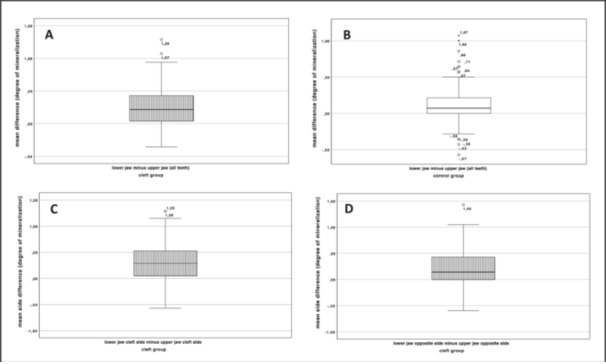
(A) Mean difference between all teeth of the lower jaw and all teeth of the upper jaw in the cleft group; mean difference: 0.25 degrees of mineralization; *p* < 0.001. (B) Mean difference between all teeth of the lower jaw and all teeth of the upper jaw in the control group; mean difference: 0.11 degrees of mineralization; *p* < 0.001. (C) Mean difference between the cleft side of the lower jaw and the cleft side of the upper jaw in the cleft group; mean difference: 0.30 degrees of mineralization; *p* < 0,001. (D) Mean difference between the opposite side of the lower jaw and the opposite side of the upper jaw in the cleft group; mean difference: 0.20 degrees of mineralization; *p* < 0.001.

In the cleft group (*n* = 180), the mean values were calculated for both the maxilla and the mandible. The mean value of the maxilla was 6.49 ± 0.97 degrees of mineralization, with a 95% CI (6.34–6.63), and that of the mandible was 6.74 ± 0.84 degrees of mineralization, with a 95% CI (6.61–6.86) (see Figure 3A). The mean difference was therefore 0.25 degrees of mineralization, with a 95% CI (0.21–0.29). The paired *t*‐test showed a significant result (*p* < 0.001).

However, a similar finding was also obtained for the control group, in which the mean degree of mineralization of the maxilla was also significantly lower than that of the mandible (maxilla: 6.81 ± 0.95, with a 95% CI (6.67–6.95), vs. mandible: 6.92 ± 0.83, with a 95% CI (6.80–7.04), *p* < 0.001) (see Figure 3B). The mean difference was 0.11 degrees of mineralization, with a 95% CI (0.08–0.15). The mean degree of mineralization of the maxilla is significantly lower than that of the mandible. To investigate in more detail whether only the cleft side shows an altered ratio of tooth mineralization between the maxilla and the mandible, mean values were obtained for the mineralization of the maxilla and the mandible on the cleft side and these were compared with each other. The mean value of the maxilla was 6.45 ± 0.97 degrees of mineralization, with a 95% CI (6.30–6.59), and that of the mandible was 6.76 ± 0.84 degrees of mineralization, with a 95% CI (6.62–6.87) (see Figure 3C). The mean difference was therefore 0.30 degrees of mineralization, with a 95% CI (0.25–0.35). The t‐test for dependent samples yielded a significant result (*p* < 0.001).

The opposite side showed slightly higher values (*p* < 0.001). The mean value of the upper jaw of the opposite side was 6.52 ± 0.99 degrees of mineralization, with a 95% CI (6.38–6.67), and that of the lower jaw was 6.73 ± 0.85 degrees of mineralization, with a 95% CI (6.60–6.85) (see Figure 3D). The mean difference was therefore 0.20 degrees of mineralization, with a 95% CI (0.16–0.25).

### 3.5 Position of the Lateral Incisors

Sometimes, several radiographs of a patient were used with a minimum interval of 24 months; therefore, the number of patients examined with cleft formation was 133. Of the 133 patients, 69 (51.9%) showed aplasia of the lateral incisor. In 29 cases (21.8%), the tooth erupted mesial to the cleft, and in 24 cases (18.0%), the tooth erupted distal to the cleft. In 11 of 133 (8.3%) patients, the tooth was duplicated. Considering only the position of the lateral incisor, the distribution ratio was 54.7% (29) for the mesial position and 45.2% (24) for the distal position.

### 3.6 Sensitivity Analysis

The results of the sensitivity analysis showed no differences compared to the primary sample, confirming the robustness of the findings. Therefore, the full cohort of 360 patients was used for subsequent analyses, emphasizing the high quality and reliability of the results in this study.

## 4 Discussion

In this study, we analyzed 180 radiographs to demonstrate a delay in tooth development in patients with cleft formation, particularly affecting the cleft side and the upper jaw more than the opposite side and the lower jaw. We used the Willems method for assessing dental age, as it proved to be the most accurate, resulting in the least under‐ or overestimation. Panoramic radiographs were chosen due to their sufficient detail and lower radiation exposure compared to CBCT. Additionally, the justifying indication is much easier to establish.

Our sample size (*n* = 180) is extensive compared to that of other studies and reflects a typical demographic distribution: more boys than girls (68.3% vs. 31.7%), with most having a complete cleft lip and palate (88.9%) and a higher occurrence on the left side (63.9%). These findings are consistent with the existing literature (Mossey et al. 2009; Hopkins et al. 2019; Voigt et al. 2017).

Previous studies often did not differentiate between types of clefts (Bindayel et al. 2014; Ranta 1982; Pham et al. 1997; Eerens et al. 2001), which can lead to inaccuracies. Our study focused on UCLP and UCLA to minimize confounding factors and facilitate comparison with future studies. We ensured uniform Caucasian ancestry in both the cleft and control groups to account for ethnic variations in tooth development.

Unlike many studies that lacked control groups or used unmatched controls (Bindayel et al. 2014; Ranta 1982; Pöyry et al. 1989), our study used an age‐, gender‐, and ethnicity‐matched control group to provide more accurate conclusions. We also conducted a sensitivity analysis with a secondary sample (*n* = 310) to confirm the robustness of our matching criteria, supporting the comprehensive evaluation of 360 patients.

### 4.1 Tooth Development

The delay in tooth development observed in our study (0.69 years) aligns with the literature, which reports delays ranging from 0.30 to 0.70 years. Only three studies did not find significant results in this area (Eerens et al. 2001; Topolski et al. 2014; Cesur et al. 2020). A study by Bindayel et al. (2014) yielded similar results (0.7 years vs. 0.69 years) but included patients of non‐Caucasian origin with different cleft types and lacked a control group. Pham et al. (1997) found similar results for boys but not for girls, and their sample size was smaller. Lai et al.‘s (Lai et al. 2008) study also showed a delay in tooth development, though it involved Chinese children. The comparable findings across these studies suggest that delayed tooth development could be a universal phenomenon in patients with cleft lip and palate. However, in addition to ethnicity, socioeconomic factors also play a significant role, and these can vary greatly between countries. Therefore, to minimize potential errors, narrowing the focus to a specific ethnic group and location, as done in our study, provides more precise results.

Our findings also align with studies conducted in other regions. For example, Van Van Dyck et al. (2021), in a study that also focused on unilateral cleft lip and palate patients, found significant delays in tooth development for patients with unilateral cleft and palate. This study examined patients aged 6–20 years individually, with the largest delays observed in 13‐year‐old girls (1.411 years) and 12‐year‐old boys (0.776 years). Although this study had a larger sample size of 189 patients, it was not without limitations, such as missing data on key tooth stages. Our study controlled for such factors by excluding patients with missing teeth and ensuring independent evaluations by two reviewers.

However, our study differs from others in that we exclusively examined Caucasian patients, allowing for more precise comparisons, but limiting our findings to a particular population. Studies involving different ethnic groups might reveal distinct patterns of tooth development, and further research is needed to confirm if our findings are generalizable to non‐Caucasian populations.

### 4.2 Asymmetrical Tooth Development

Past studies on asymmetric tooth development in patients with cleft lip and palate have shown varying results due to differences in methods and ethnicities. Our study aimed to investigate this in a large population. We found significant asymmetry in tooth development (*p* = 0.001), with a mean difference of 0.08 developmental stages (Demirjian method) between the cleft side and the opposite side. Significant delays were observed in the canine and lateral and central incisors. The maxilla was also lagged behind the mandible by 0.25 stages in the cleft group. Similarly, in the control group, the maxilla lagged behind the mandible, but the difference was smaller (0.11 stages). We found a significant difference in tooth development between the upper and lower jaws on both the cleft and opposing sides (cleft side mean difference: 0.30 vs. opposite side mean difference: 0.20, *p* < 0.001).

Comparing our results with the existing literature, we found that only a few studies used the Demirjian method, including Eerens et al. (2001), Tan et al. (2018, 2017), Lai et al. (2008), and Van Van Dyck et al. (2021). Eerens et al. (2001) also found asymmetric tooth development but did not compare both jaws. Lai et al. (2008) observed the most pronounced development delay in the lateral incisor in a group of Chinese patients with various cleft types. Tan et al. (2017) reported an increased risk of asymmetric tooth development in cleft patients, with the lateral incisor most frequently affected and the maxilla lagging behind the mandible.

In the study by Van Van Dyck et al. (2021), which is methodologically closest to our approach, a mean difference of 0.03 in girls and 0.04 in boys was observed in 189 patients. In addition, a slight delay of the maxilla to the mandible was also observed, which is consistent with our results. A comparison of the upper and lower jaw between the cleft side and the opposite side did not provide a significant result. However, a limitation of their study was the restriction of the analysis to a single examiner. In our study, this examiner‐dependent bias was minimized by performing two independent analyses.

### 4.3 Lateral Incisor

Due to multifactorial embryological, anatomical, and environmental factors, the maxillary lateral incisor is particularly prone to developmental abnormalities, often resulting in its absence, a phenomenon commonly reported in the literature (Tan et al. 2018; Lai et al. 2009; Jamal et al. 2010). Therefore, our study also explores the positioning of the lateral incisor relative to the cleft. It can be found mesial or distal to the cleft, completely absent, or even duplicated. The most frequent observation is aplasia of the lateral incisor (52.28%). When present, it is more commonly located mesial (21.96%) rather than distal (17.42%) to the cleft. Double existence of the lateral incisor was observed less frequently (8.33%).

The literature reports similar occurrences of tooth aplasia, ranging from 12.4% to 79.2% in the permanent dentition on the cleft side, with variations observed across continents and genders. Studies by Tsai et al. (1998) (51.8%), Ribeiro et al. (2002) (49.8%), and Tortora et al. (2008) (48.8%) show comparable results. Dental aplasia was noted to be higher in Europe and Australia compared to North American Caucasians, with a 1.37 times higher prevalence in females (Polder et al. 2004). Antonarakis et al.‘s study (Antonarakis et al. 2021) observed similar values in Caucasian patients (46.4% vs. 52.2%).

The prevalence of a double lateral incisor in our study aligns with previous findings. Regarding its positioning, although the literature often reports a higher frequency of the distal position, our results differ (mesial 54.7%, distal 45.2%) (Tsai et al. 1998; Keith 1909; Pegelow et al. 2012). Methodological consistency rules out analysis differences as the cause. In addition to the heterogeneity of the cleft types and ethnicities in some studies, gentler operations and a different orthodontic treatment (with preservation of the hypoplastic lateral incisor) can also be considered as possible explanations for the different results. According to the hypothesis of Tan et al. (2018), the primary palate is formed by the fusion of the maxilla and the medial nasal projection and the primary palate is part of the intermaxillary segment, which, in addition to the philtrum, consists of the adjacent central part of the maxilla with its four anterior teeth. Splitting the primary palate should result in the lateral incisor being positioned mesial to the cleft, as the lateral incisor is part of the intermaxillary segment. Recent evidence seems to indicate that there is a double embryonic origin of the maxillary lateral incisors, with the mesial half originating from the medial nasal process and the distal half originating from the maxillary process (Tsai et al. 1998; Tan et al. 2018; Hovorakova et al. 2006; Wei et al. 2000). According to Tsai et al. (1998), the failure of the two processes to fuse could be the cause of the development of different distribution patterns (Tsai et al. 1998; Tan et al. 2018).

### 4.4 Study Limitations

The study has several limitations. A selection bias may arise from the sample being drawn from a single center, which could limit the generalizability of the findings. The panoramic radiographs used are less precise than CBCT, which could lead to inaccuracies in mineralization assessment. Additionally, the asymmetry in dental development could be influenced by factors such as treatment methods or early maxillofacial surgery. Furthermore, the study period, with some patients being examined with more than 24 months between radiographs, might affect the accuracy of developmental assessment. A longer observation period could help to reduce these inaccuracies.

Despite these potential limitations, it is important to emphasize that the errors in our study are overall very minimal due to the targeted nature of the examinations. Two different, independent examiners assessed the stages, and key factors such as age, ethnicity, and cleft types were precisely defined and controlled. This methodological rigor ensures a high level of validity and accuracy in our study compared to others.

## 5 Conclusion

For orthodontic tooth movement to proceed without tissue damage, a precisely calibrated force is required—sufficient to enable tooth movement but gentle enough to avoid harming the periodontal tissues (2nd Biological Effectiveness according to A.M. Schwarz) (Wichelhaus 2013). The calculation of the optimal force takes into account the root surface area, which is smaller in teeth with incomplete root development (Wichelhaus 2013). Therefore, orthodontic treatment should commence only after full root development to prevent potential damage. This is particularly relevant for young patients with unilateral cleft lip and palate, where root development on the cleft side has been shown to be delayed, making a later treatment start beneficial.

## Author Contributions

The preparation and conception of the study, as well as data collection, were carried out by Marie Schwarting and Susanne Wriedt. The statistical analysis was performed by Irene Schmidtmann and Marie Schwarting. The manuscript was written by Marie Schwarting. All authors reviewed the manuscript, provided feedback on the first and second versions, and approved the final version.

## Conflicts of Interest

The authors declare no conflicts of interest.

## Data Availability

The data that support the findings of this study are available from the corresponding author upon reasonable request.
